# Profiles of affective temperaments can support differentiation of bipolar I disorder, bipolar II disorder, and major depressive disorder

**DOI:** 10.1192/j.eurpsy.2025.1069

**Published:** 2025-08-26

**Authors:** M. Cho, C. H. K. Park

**Affiliations:** 1 Psychology, Seoul National University; 2 Psychiatry, Asan Medical Center, Seoul, Korea, Republic Of

## Abstract

**Introduction:**

Differentiating bipolar I, bipolar II, and major depressive disorders is essential; therefore, the relationship between affective temperaments and mood disorder diagnoses has garnered considerable attention.

**Objectives:**

The current study aimed to explore the representative types of Temperament Evaluation of Memphis, Pisa, Paris, and San Diego-Autoquestionnaire (TEMPS-A) profiles observed in the actual clinical setting using a data-driven approach and verify the relationship between TEMPS-A profiles and the proportions of three mood disorders (bipolar I, bipolar II, and major depressive disorders). Through this research, we intend to enhance the utility and applicability of affective temperament assessment based on the TEMPS-A when differentiating among mood disorders in clinical practice.

**Methods:**

Psychiatric outpatients diagnosed with bipolar I, bipolar II, or major depressive disorder and aged ≥18 years were analyzed. Latent profile analysis was conducted using TEMPS-A scores, and each patient was classified into a subgroup according to their TEMPS-A scores. After that, multinomial logistic regression was conducted to verify the relationship between TEMPS-A profiles and mood disorder diagnoses.

**Results:**

The findings indicate that seven types of TEMPS-A profiles are the most appropriate (Table 1). The depressive profile did not significantly increase the likelihood of being diagnosed with both bipolar II disorder and bipolar I disorder. The cyclothymic profile and anxious, cyclothymic and depressive profiles significantly increased the likelihood of the diagnosis of bipolar II disorder but did not significantly increase the likelihood of the diagnosis of bipolar I disorder. Furthermore, the cyclothymic and hyperthymic profile; cyclothymic, depressive and irritable profile; and anxious, cyclothymic, hyperthymic and irritable profiles significantly augmented the likelihood of being diagnosed with both bipolar II disorder and bipolar I disorder (Tables 2 and 3).

**Image 1:**

**Figure:**
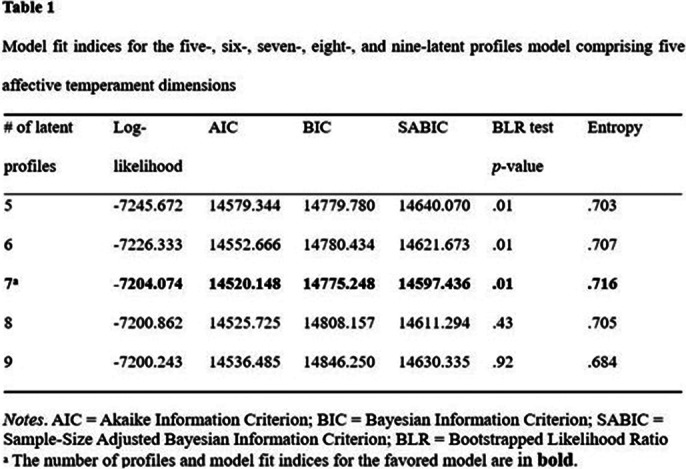

**Image 2:**

**Figure:**
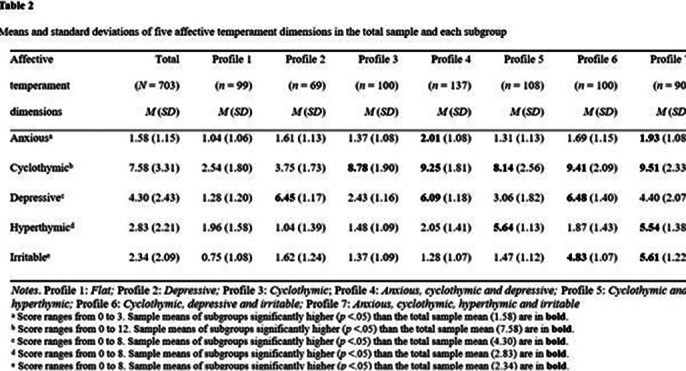

**Image 3:**

**Figure:**
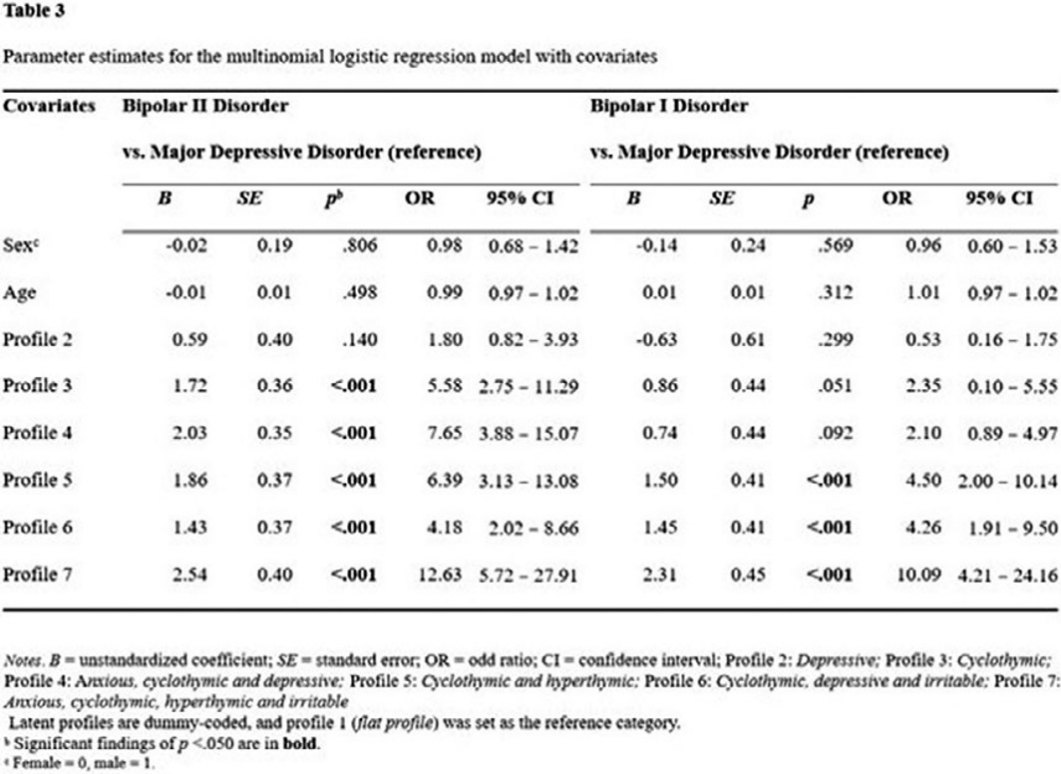

**Conclusions:**

Our findings show that certain affective temperaments are closely correlated with one another, and cyclothymic temperament can act as a crucial risk factor for bipolarity. Moreover, our findings suggest the possibility that hyperthymic or irritable temperaments can help differentiate between bipolar I disorder and bipolar II disorder. Further studies using reliable clinical interview schedules are needed, and studies focusing on patients with other mood disorders would be helpful.

**Disclosure of Interest:**

None Declared

